# DDR1 Modulates Cytoskeletal Remodeling and Podosome Formation in Renal Fibroblasts

**DOI:** 10.3390/ijms27125419

**Published:** 2026-06-16

**Authors:** Po-Yu Chen, Gang-Hui Lee, Yi-Chun Yeh, Chia-Jung Chang, Chao-Kai Hsu, Ming-Jer Tang

**Affiliations:** 1 Institute of Basic Medical Sciences, College of Medicine, National Cheng Kung University, Tainan 701, Taiwan; s58114053@gs.ncku.edu.tw; 2 International Center for Wound Repair and Regeneration, National Cheng Kung University, Tainan 701, Taiwan; 3 Department of Physiology, College of Medicine, National Cheng Kung University, Tainan 701, Taiwan; 4 Department of Physiology and Pharmacology, Graduate Institute of Biomedical Sciences, Chang Gung University, Taoyuan 333, Taiwan; 5 Kidney Research Center, Chang Gung Memorial Hospital, School of Medicine, Chang Gung University, Taoyuan 333, Taiwan; 6 Department of Dermatology, National Cheng Kung University Hospital, College of Medicine, National Cheng Kung University, Tainan 701, Taiwan; 7 Institute of Clinical Medicine, College of Medicine, National Cheng Kung University, Tainan 701, Taiwan

**Keywords:** DDR1, fibroblast, mechanobiology, podosome, kidney fibrosis

## Abstract

Discoidin domain receptor 1 (DDR1) has been implicated in fibrotic progression in multiple organs, including the kidney. However, its role in regulating cytoskeletal organization and matrix remodeling in renal fibroblasts remains unclear. Here, we investigated how DDR1 expression is regulated by profibrotic stimulation and extracellular matrix stiffness, and how DDR1 influences cytoskeletal organization and collagen remodeling. Single-cell RNA sequencing of murine kidneys subjected to unilateral ureteral obstruction (UUO) revealed enrichment of Ddr1 expression in transitional fibroblast populations during early activation. In vitro, transforming growth factor-β1 (TGF-β1) increased DDR1 expression, but DDR1 depletion did not affect canonical myofibroblast marker expression. Instead, DDR1 depletion suppressed stress fiber assembly while promoting actin-rich podosome formation associated with matrix degradation. Functionally, DDR1-deficient cells exhibited impaired focal adhesion maturation, enhanced collagen degradation, reduced gel contraction, and decreased collagen matrix stiffness as measured by atomic force microscopy. Furthermore, extracellular matrix stiffness dynamically regulated DDR1 expression, suggesting a bidirectional relationship between DDR1 expression and matrix mechanics. Together, these findings identify DDR1 as a modulator of cytoskeletal remodeling that governs the balance between matrix-degradation and contractile remodeling programs in renal fibroblasts.

## 1. Introduction

Renal fibrosis is a hallmark of chronic kidney disease and is characterized by excessive accumulation and remodeling of extracellular matrix (ECM), ultimately leading to progressive loss of renal function. Fibroblasts are highly plastic mesenchymal cells that can adopt distinct phenotypic states in response to environmental cues [1,2]. During fibrotic progression, activated fibroblasts and myofibroblasts produce large amounts of ECM proteins, for example type I collagen and fibronectin, primarily driven by profibrotic signaling pathways including transforming growth factor-β (TGF-β) [3,4,5]. In addition to matrix production, activated fibroblasts exert contractile forces that reorganize collagen fibers and increase tissue stiffness, establishing a reinforcing mechanical and biochemical feedback loop that contributes to fibrotic progression [6,7,8,9].

While fibroblast activation and matrix deposition have been extensively studied, comparatively less is known about how fibroblasts regulate matrix degradation during fibrogenesis [10,11]. Actin-rich podosomes are specialized adhesion structures capable of mediating localized ECM degradation through the recruitment of matrix metalloproteinases [12,13,14]. Although podosome formation has been well characterized in macrophages, dendritic cells, and osteoclasts [15,16], increasing evidence indicates that fibroblasts can also assemble podosome-like structures under specific biochemical and mechanical conditions [17]. However, the regulatory mechanisms and functional significance of podosome formation in fibroblasts remain poorly understood.

Unilateral ureteral obstruction (UUO) is one of the most widely used experimental models of renal fibrosis and is characterized by progressive tubular injury, inflammatory cell infiltration, fibroblast activation, and excessive extracellular matrix deposition. Previous studies have shown that collagen receptors, including integrins and discoidin domain receptors (DDRs), participate in the regulation of fibroblast behavior and tissue remodeling during UUO-associated fibrosis [18,19]. In particular, DDR1 deficiency attenuates renal inflammation and fibrosis following UUO [20], whereas the specific roles of DDR family members in fibroblast remodeling responses remain incompletely understood.

Discoidin domain receptor 1 (DDR1) is a collagen-activated receptor tyrosine kinase that regulates cytoskeletal organization and cell–matrix interactions [21]. Unlike integrins, which function as primary cell adhesion receptors mediating cell–extracellular matrix interactions [22,23], DDRs belong to a family of collagen-activated receptor tyrosine kinases that primarily function as signaling receptors to regulate cell–matrix interactions. Although DDR2 is generally considered the predominant discoidin domain receptor in fibroblasts under physiological conditions, DDR1 expression can be induced under pathological or activated states. It has been implicated in fibrotic diseases across multiple organs, including the kidney, where DDR1 signaling contributes to inflammation and fibrotic progression [20,24]. Recent studies have suggested that DDR1 may participate in cellular responses to mechanical cues such as extracellular matrix stiffness [25]. However, its role as a direct mechanosensor remains under investigation and is supported by limited evidence.

In this study, we investigated how DDR1 expression is regulated by profibrotic signaling and matrix stiffness and how DDR1 influences cytoskeletal organization and matrix remodeling in renal fibroblasts. By integrating single-cell transcriptomic analysis of fibrotic kidneys with mechanobiological assays in vitro, we show that DDR1 is associated with a contractile cytoskeletal state and inversely correlates with podosome formation. Taken together, this research supports a role for DDR1 in regulating the balance between matrix-degradative and contractile remodeling programs.

## 2. Results

### 2.1. Single-Cell Transcriptomic Profiling Defines Fibroblast State Transitions and State-Specific Ddr1 Expression During UUO Progression

**Figure 1:**
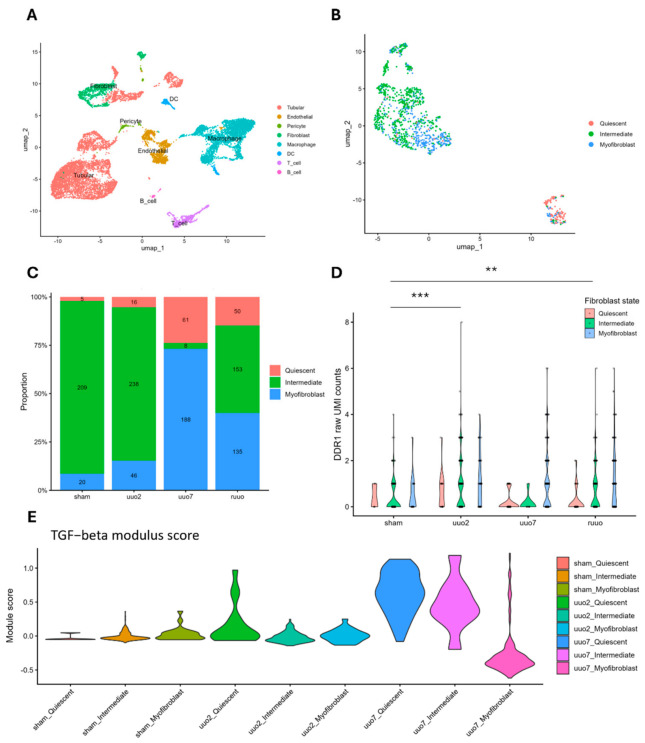
Single-cell landscape of renal fibroblast states and *Ddr1* expression during injury and recovery. (**A**) UMAP projection of integrated single-cell RNA-seq data from sham, UUO 2 (uuo2), UUO 7 (uuo7), and reversal UUO (ruuo) kidneys. Major renal cell populations were annotated based on canonical marker gene signatures, including tubular epithelial cells, endothelial cells, pericytes, fibroblasts, macrophages, dendritic cells (DC), T cells, and B cells. (**B**) Re-clustering and UMAP visualization of fibroblast populations extracted from the integrated dataset. Fibroblasts were classified into three transcriptional states—Quiescent, Intermediate, and Myofibroblast—based on module scores derived from quiescent-associated, ECM-remodeling, and contractile gene signatures. (**C**) Relative distribution of fibroblast states across experimental conditions. Stacked bar plots show the proportion of Quiescent (red), Intermediate (green), and Myofibroblast (blue) states within each condition. Numbers within bars indicate cell counts per state. (**D**) Mean *Ddr1* raw UMI counts across fibroblast states and conditions. Bars represent mean ± SEM. Statistical comparisons were performed within each fibroblast state using Wilcoxon rank-sum tests with Benjamini–Hochberg correction (vs. sham). (**E**) TGF-β response module scores across fibroblast states and experimental conditions. Violin plots show the distribution of module scores in each state–condition group, reflecting the activation of profibrotic signaling programs. ** *p* < 0.01; *** *p* < 0.001.

To characterize fibroblast heterogeneity during kidney injury and recovery, we analyzed single-cell RNA sequencing data from sham, UUO day 2 (UUO 2), UUO day 7 (UUO 7), and reversed UUO (rUUO) kidneys (Figure 1A). We employed canonical marker annotation to identify major renal cell populations, including tubular epithelial cells, endothelial cells, pericytes, fibroblasts, and immune subsets. Fibroblasts were extracted and re-clustered to resolve intrapopulation heterogeneity (Figure 1B). Based on curated gene signatures, fibroblasts were classified into three transcriptional states: quiescent, intermediate, and myofibroblast. Marker gene expression supported this classification (Figure S1A), with quiescent fibroblasts enriched for Pdgfra, Dcn, and Lum, and myofibroblasts expressing elevated levels of Acta2, Tagln, Col1a1, and Postn. Module score analysis identified distinct enrichment of quiescent, ECM, and contractile programs across the three states (Figure S1B), with intermediate fibroblasts exhibiting transitional features. This fibroblast state distribution shifted according to condition (Figure 1C), with expansion of activated populations during UUO and partial restoration after reversal. We next examined Ddr1 expression across fibroblast states and injury conditions. Quantification of raw UMI counts revealed state-dependent differences (Figure 1D). Ddr1 expression was significantly increased within the intermediate fibroblast population at UUO 2 and rUUO compared with sham. No significant differences were detected in quiescent or fully differentiated myofibroblast states. Because fibroblast states were defined within the same integrated dataset, the observed increase in Ddr1 expression reflects transcriptional changes within transitional fibroblast states. This change was unlikely to result from differences in cell-type composition. To further assess whether these fibroblast states were associated with profibrotic activation, we examined the TGF-β response module scores across fibroblast states and conditions (Figure S1C). TGF-β response module scores increased in activated fibroblast populations during UUO, particularly at UUO 2, consistent with enhanced profibrotic activation during early injury. Notably, TGF-β response scores were reduced in UUO 7 myofibroblasts compared with earlier stages, suggesting that mature myofibroblast states may not fully retain the transcriptional features of an early TGF-β response program (Figure 1E).

### 2.2. TGF-β1 Induces DDR1 Upregulation During Myofibroblast Activation

**Figure 2:**
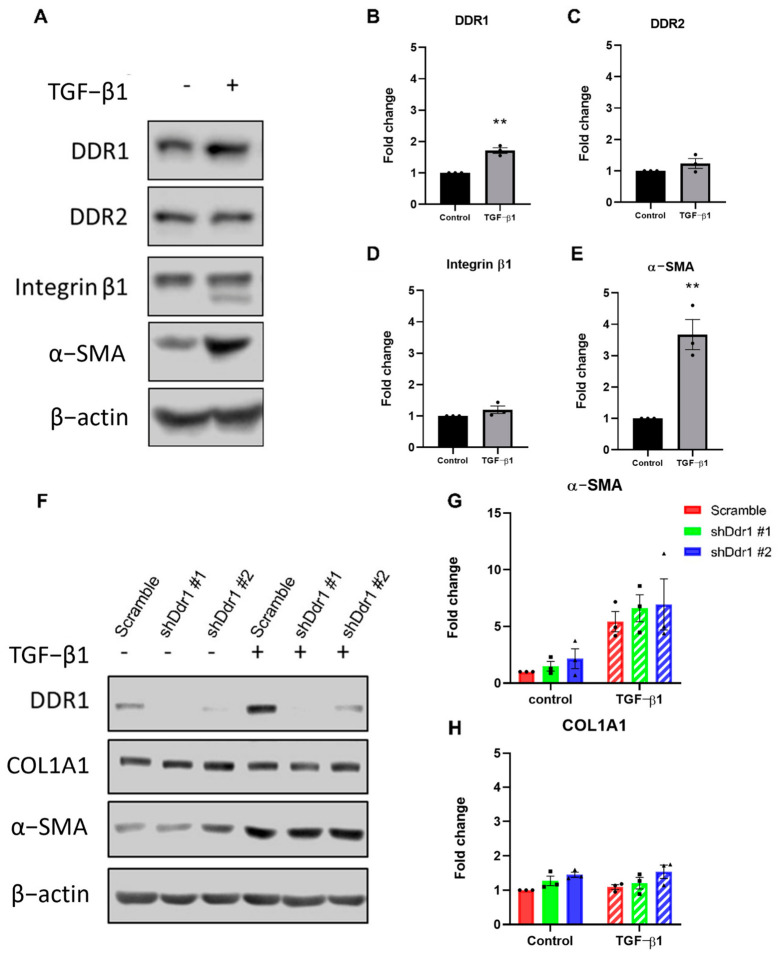
TGF-β1 induces upregulation of DDR1 during myofibroblast activation. (**A**) Western blot analysis of collagen receptors and α-SMA in NRK-49F cells treated with or without TGF-β1 (10 ng/mL, 24 h). (**B**–**E**) Quantification of DDR1, DDR2, total integrin β1, and active integrin β1 expression levels (*n* = 3). (**F**–**H**) Scramble control and DDR1 knockdown (shDdr1) NRK-49F cells were treated with or without TGF-β1 for 24 h. Cell lysates were analyzed for α-SMA and COL1A1 expression by Western blot (*n* = 3). Densitometric analysis was performed using ImageJ (version 1.54p). Statistical analyses were performed using Student’s *t* test (**B**–**E**) or two-way ANOVA (**G**,**H**) with Prism 8 software. Error bars represent mean ± SEM. ** *p* < 0.01.

Given the injury-associated upregulation of Ddr1 in activated fibroblast states in vivo, we next asked whether canonical profibrotic stimulation similarly regulates DDR1 expression in vitro. NRK-49F cells were treated with transforming growth factor-β1 (TGF-β1), a well-established driver of myofibroblast activation [3,26]. Western blot analysis demonstrated DDR1 protein levels were significantly increased following TGF-β1 treatment (Figure 2A,B), whereas DDR2 expression remained unchanged (Figure 2A,C). Total integrin β1 levels showed a modest increase upon TGF-β1 stimulation (Figure 2A). In parallel, α-smooth muscle actin (α-SMA) was markedly upregulated by TGF-β1 (Figure 2A,E). Because DDR1 expression was elevated during TGF-β1-induced activation, we next tested whether DDR1 was required for myofibroblast differentiation. DDR1 depletion did not significantly alter TGF-β1-induced upregulation of α-SMA or change COL1A1 expression compared with scramble controls (Figure 2F–H), indicating that DDR1 is dispensable for TGF-β1-driven myofibroblast marker induction under these conditions.

### 2.3. DDR1 Depletion Promotes Podosome Formation and Suppresses Stress Fiber Assembly

**Figure 3:**
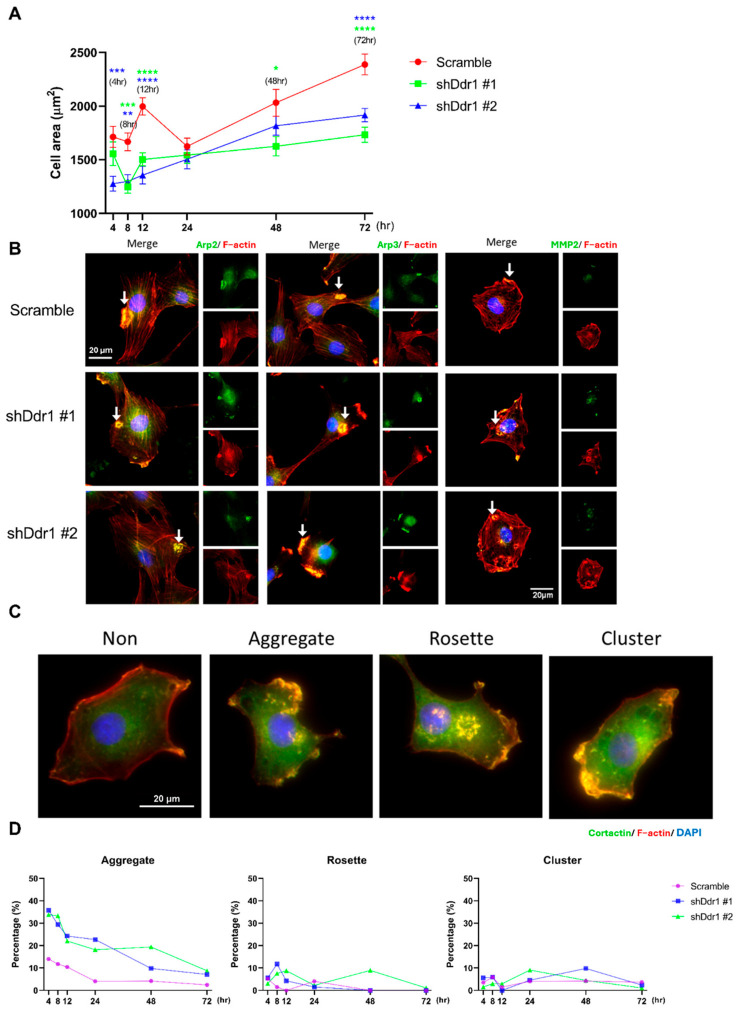
DDR1 knockdown restricts cell spreading and promotes podosome aggregate formation. (**A**) Cell spreading area was quantified over time in scramble and shDdr1 cells cultured on tissue culture dishes. Error bars represent mean ± SD. * *p* < 0.05; ** *p* < 0.01; *** *p* < 0.001; **** *p* < 0.0001. (**B**) Representative immunofluorescence images showing actin aggregates colocalized with podosome markers (Arp2/3, cortactin, and MMP2) after 24 h of culture. White arrows indicate actin-rich aggregates. (**C**) Representative images illustrating distinct podosome organizational patterns: aggregates, rosettes, and clusters. Blue fluorescence indicates DAPI in all images. (**D**) Quantification of podosome subtypes over time. The quantified data represent the distribution of cellular phenotypes obtained from a single experiment, in which 50–80 cells per condition were analyzed and classified according to the indicated criteria.

**Figure 4:**
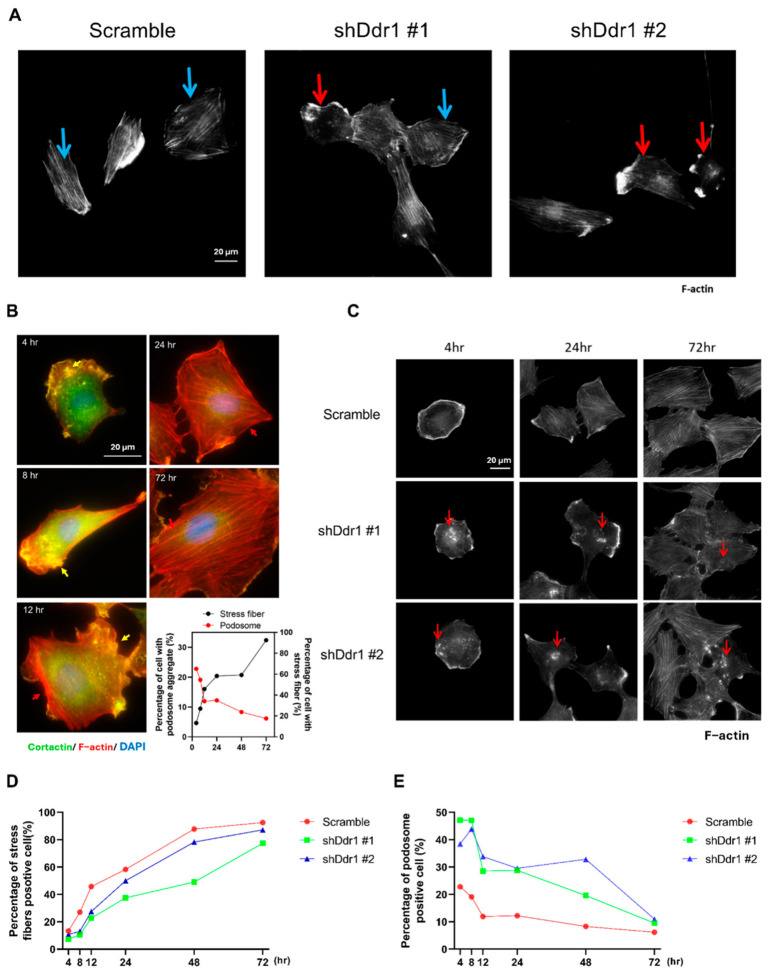
DDR1 depletion suppresses stress fiber assembly and enhances podosome formation. (**A**) Representative F-actin staining images of scramble and shDdr1 cells after 24 h in culture. Blue arrows indicate stress fiber-positive cells; red arrows indicate podosome-positive cells. (**B**) Time-course analysis of actin organization in NRK-49F cells. Yellow arrows indicate podosomes; red arrows indicate stress fiber. (**C**) Representative F-actin staining images. (**D**,**E**) Quantification of stress fiber-positive and podosome-positive cells. The quantified data represent the distribution of cellular phenotypes obtained from a single experiment, in which 50–80 cells per condition were analyzed and classified according to the indicated criteria.

Fibroblast-mediated matrix remodeling is closely associated with changes in cell morphology and actin cytoskeletal organization. Because DDR1 depletion did not alter TGF-β1-induced myofibroblast marker expression, we next examined whether DDR1 influences fibroblast structural remodeling independently of canonical differentiation programs. To address this question, cell spreading dynamics and actin organization were analyzed in control and DDR1-deficient NRK-49F cells. DDR1-deficient NRK-49F cells exhibited reduced cell spreading over time compared with control cells (Figure 3A), indicating altered cytoskeletal organization. Concomitantly, prominent actin-rich aggregates were frequently detected in DDR1-deficient cells. Immunofluorescence analysis revealed that these structures colocalized with established podosome markers, including Arp2/3, cortactin, and MMP2 (Figure 3B), confirming their identity as podosome-like assemblies. Podosomes assembled into distinct organizational patterns [12], including aggregates, rosettes, and clusters (Figure 3C). Quantitative analysis showed that DDR1 knockdown selectively increased the prevalence of podosome aggregates, whereas rosette and cluster formation were not significantly altered (Figure 3D). Strikingly, stress fiber-positive cells rarely exhibited podosome structures, and conversely, podosome-positive cells lacked prominent stress fibers, revealing a mutually exclusive distribution of these two actin architectures at both the single-cell and population levels (Figure 4A,B). DDR1 depletion shifted the population balance toward increased podosome-positive cells and decreased stress fiber-positive cells (Figure 4C–E), suggesting that DDR1 normally promotes a contractile actin organization while suppressing matrix-degradative podosome formation.

### 2.4. DDR1 Is Associated with Focal Adhesion Complex Maturation

**Figure 5:**
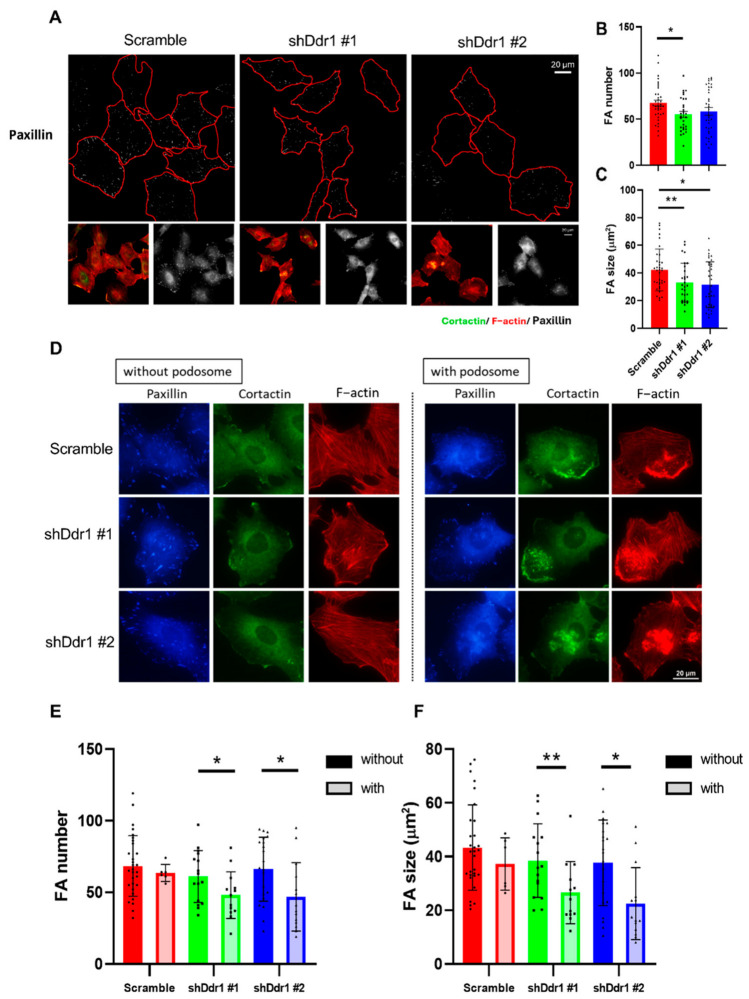
DDR1 depletion reduces focal adhesion size and number. (**A**) Representative immunofluorescence images of F-actin, paxillin (focal adhesions), and cortactin (podosomes) in scramble and shDdr1 cells cultured for 24 h. For image presentation, convolution filtering was applied to paxillin images to reduce background noise. Scale bars = 20 µm. (**B**,**C**) For focal adhesion quantification, focal adhesion number and size were measured using the original raw images. Convolution filtering was applied only for image visualization and was not used for quantitative analysis (*n* = 3). (**D**–**F**) Stratified analysis of focal adhesion parameters in podosome-negative and podosome-positive cells (*n* = 3). Statistical analyses were performed using one-way or two-way ANOVA with Prism 8 software. Error bars represent mean ± SD. * *p* < 0.05; ** *p* < 0.01.

Because focal adhesions coordinate cytoskeletal organization and cell–matrix adhesion, we next examined whether DDR1 regulates focal adhesion organization. Quantitative analysis revealed both the number and total area of paxillin-positive focal adhesions were significantly reduced in DDR1 knockdown cells compared with scramble controls (Figure 5A–C), consistent with impaired focal adhesion maturation. To determine whether focal adhesion alterations were linked to actin state transitions, cells were stratified according to the presence or absence of podosome structures. In podosome-negative cells, focal adhesion number and size were comparable between control and DDR1 knockdown groups (Figure 5D–F). In contrast, among podosome-positive cells, DDR1 depletion was associated with a marked reduction in both focal adhesion number and size, indicating DDR1 contributed to adhesion maturation in cells undergoing podosome-associated cytoskeletal remodeling.

### 2.5. Matrix Stiffness Dynamically Regulates DDR1 Expression and Podosome Formation

**Figure 6:**
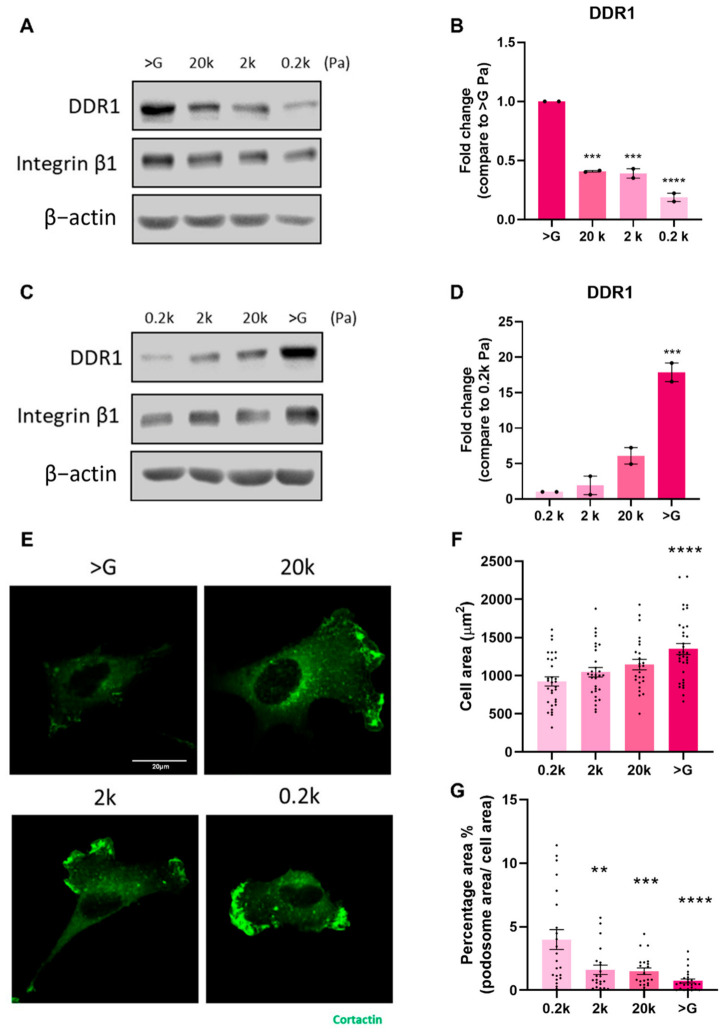
Matrix stiffness regulates DDR1 expression and podosome formation. NRK-49F cells were cultured on tissue culture plastic (>GPa) or collagen-coated polyacrylamide (PA) gels with defined stiffness (20 kPa, 2 kPa, or 0.2 kPa). (**A**,**C**) Western blot analysis of DDR1 and integrin β1 expression under different stiffness conditions. (**B**,**D**) Quantification of protein expression levels. For panels (**A**,**B**), cells were maintained on tissue culture plastic prior to seeding onto softer PA gels, and tissue culture plastic was used as the control condition. For panels (**C**,**D**), cells were maintained on 0.2 kPa gels prior to reseeding onto stiffer substrates, and 0.2 kPa was used as the control condition (*n* = 2). (**E**) Representative immunofluorescence images of cortactin-positive podosomes under different stiffness conditions. (**F**,**G**) Quantification of cell area and podosome area relative to total cell area (*n* = 3). Statistical analyses were performed using Student’s *t* test or one-way ANOVA as indicated. Error bars represent mean ± SEM. ** *p* < 0.01; *** *p* < 0.001; **** *p* < 0.0001.

Since progressive matrix stiffening is a defining biomechanical feature of fibrotic tissues and focal adhesion signaling is sensitive to substrate mechanics, we next examined whether extracellular matrix stiffness regulates DDR1 expression. NRK-49F cells were cultured on substrates of defined stiffness (0.2 kPa, 2 kPa, and 20 kPa) as well as on tissue culture plastic (>GPa). DDR1 protein expression was progressively reduced in cells cultured on soft substrates compared with stiff substrates (Figure 6A–D), indicating stiffness-dependent regulation of DDR1 expression. In agreement with the phenotype observed upon DDR1 depletion, culture on soft substrates was associated with increased podosome formation and reduced cell spreading (Figure 6E–G). Collectively, these data indicated reduced matrix stiffness suppressed DDR1 expression and promoted a podosome-dominant cytoskeletal state.

### 2.6. DDR1 Depletion Reduces Collagen Matrix Stiffness and Impairs Contractile Matrix Remodeling While Enhancing Collagen Degradation

**Figure 7:**
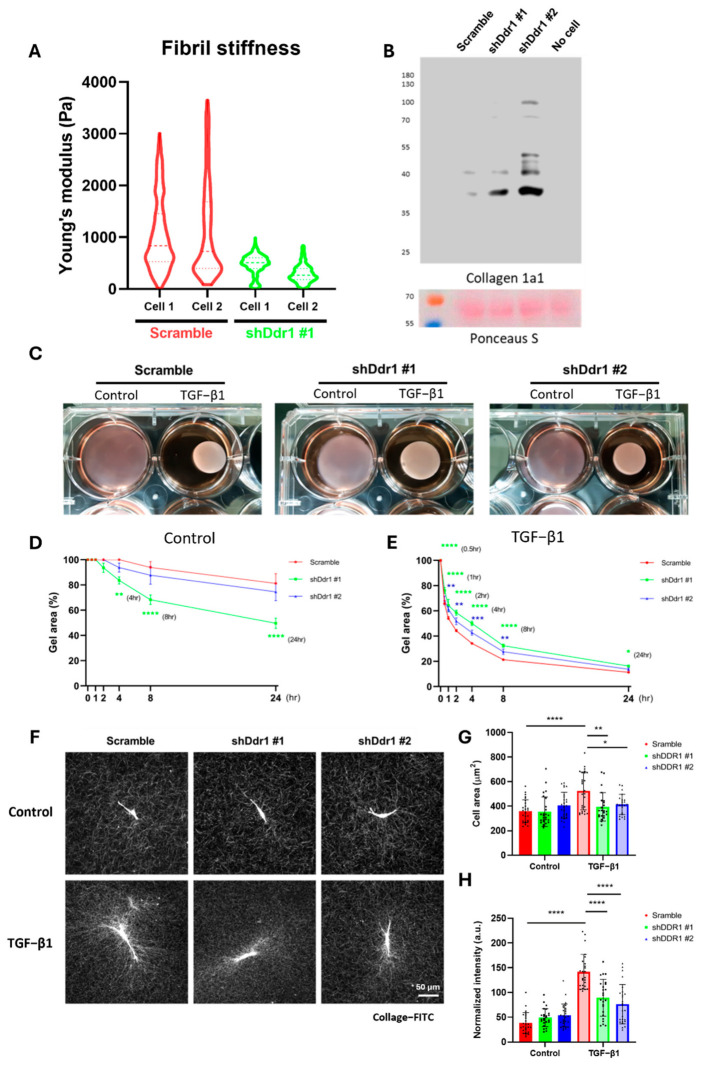
DDR1 depletion enhances collagen degradation but attenuates contractile matrix remodeling. (**A**) Atomic force microscopy (AFM) measurements of collagen fibril stiffness. NRK-49F cells expressing scramble control or shDdr1 #1 were seeded onto collagen gels, and local fiber stiffness was quantified. Representative distributions and quantification are shown (*n* = 2). (**B**) Collagen fragmentation assay showing increased low-molecular-weight collagen I fragments in conditioned media from shDdr1 cells. Ponceau S staining was used as a loading control (*n* = 2). (**C**) Representative images of collagen gel contraction assays after gel release (*n* = 4). (**D**,**E**) Quantification of gel area over time. (**F**) FITC-labeled collagen aggregation assay. Cells were stained with phalloidin to visualize cell morphology. (**G**,**H**) Quantification of cell area and extracellular FITC fluorescence intensity (normalized to background) (*n* = 3). Statistical analyses were performed using Student’s *t* test or two-way ANOVA. Error bars represent mean ± SEM. * *p* < 0.05; ** *p* < 0.01; *** *p* < 0.001; **** *p* < 0.001.

To investigate the functional consequences of DDR1 depletion on matrix remodeling, we cultured renal fibroblasts on FITC-conjugated type I collagen gel for 5 days and employed atomic force microscopy (AFM) to assess the mechanical properties of collagen fibrils remodeled by renal fibroblasts. The results of AFM measurements revealed collagen fibril stiffness was significantly reduced in DDR1 knockdown conditions compared with scramble controls (Figure 7A), indicating that DDR1 contributed to matrix stiffening. We next performed collagen degradation and gel contraction assays. When NRK-49F cells were cultured on collagen gels, increased levels of low-molecular-weight collagen I fragments were detected in the conditioned medium of DDR1 knockdown cells compared with scramble controls (Figure 7B), indicating enhanced collagen degradation. Next, collagen gel contraction assays were used to evaluate cell-mediated matrix remodeling. In the absence of TGF-β1, collagen gel area was moderately reduced in DDR1 knockdown cultures relative to controls (Figure 7C,D), consistent with altered matrix remodeling dynamics. As expected, TGF-β1 treatment markedly enhanced gel contraction in control cells. In contrast, DDR1 knockdown significantly attenuated TGF-β1-induced gel contraction (Figure 7C,E), indicating reduced contractile remodeling of the collagen matrix under profibrotic stimulation. To further assess collagen fibril organization by fibroblasts, cells were cultured in FITC-labeled collagen gels. TGF-β1 treatment increased cell spreading and collagen fibril organization in control cells, whereas these responses were significantly reduced in DDR1 knockdown cells (Figure 7F–H). Taken together, these results demonstrated that DDR1 depletion reduces collagen matrix stiffness, promotes collagen degradation, and impairs collagen fibril organization and contractile remodeling, thereby shifting fibroblasts toward a matrix-degradative and mechanically softened phenotype.

## 3. Discussion

Fibrotic progression is driven not only by profibrotic signaling but also by dynamic biomechanical changes in the extracellular matrix [27]. Although fibroblast activation and matrix deposition have been extensively studied [2,26], the mechanisms that determine whether fibroblasts adopt contractile or matrix-degradative remodeling programs remain incompletely understood. In line with prior knowledge that DDR2 is the predominant discoidin domain receptor in fibroblasts, our findings suggest that DDR1 is selectively induced under activated or profibrotic conditions. This was supported by our single-cell RNA sequencing analysis. Ddr2 expression was primarily restricted to quiescent fibroblast populations, with minimal expression in intermediate and myofibroblast states, whereas Ddr1 expression was enriched in activated fibroblast populations (Figure S1D).

**Figure 8:**
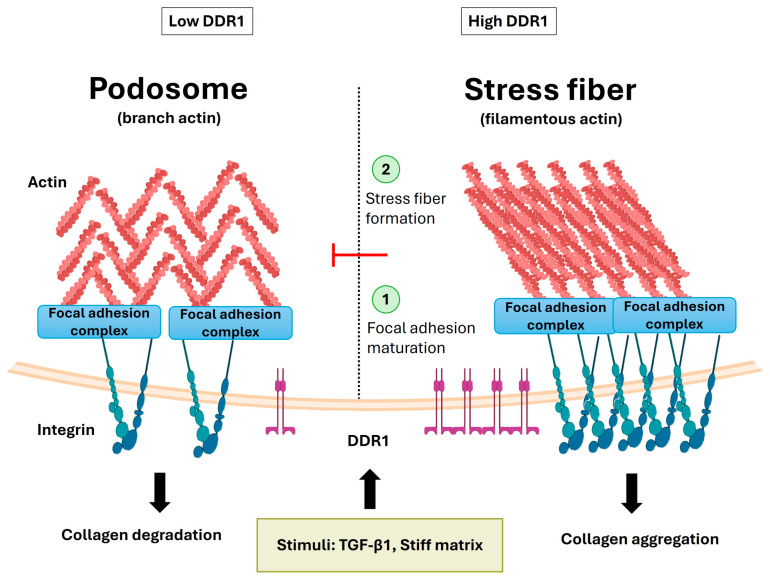
Schematic model of DDR1-regulated cytoskeletal remodeling and collagen dynamics in renal fibroblasts. Reduced DDR1 expression promotes podosome formation and collagen degradation, whereas elevated DDR1 expression favors stress fiber assembly and contractile remodeling under conditions of increased matrix stiffness. Through this mechanoresponsive regulation of cytoskeletal organization, DDR1 influences the balance between matrix-degradative and contractile remodeling programs during renal fibrogenesis. Created in BioRender. Chiu, W. (2026) https://BioRender.com/seb4pf0 (accessed on 14 May 2026).

In this study, we identify DDR1 as a modulator of mechanoregulation that influences cytoskeletal organization and matrix-remodeling behavior in renal fibroblasts. Our findings indicate that DDR1 is associated with stress fiber assembly and contractile remodeling, whereas reduced DDR1 expression is associated with increased podosome formation and collagen degradation (Figure 8), thereby influencing the balance between distinct remodeling states during fibrotic progression.

A central observation of this study is a predominantly mutually exclusive relationship between stress fiber assembly and podosome formation, which represent two alternative cytoskeletal architectures associated with contractile and matrix-degradative functions, respectively. Consistent with this concept, depletion of tropomyosin 1.6 has been shown to suppress contractile activity while promoting the formation of α-SMA–MMP9-enriched matrix-degrading structures, thereby shifting fibroblasts toward a degradative phenotype [28]. Reduced DDR1 expression, either through genetic depletion or culture on soft matrices, is associated with increased podosome formation and enhanced collagen degradation, whereas elevated DDR1 expression on stiff substrates correlates with stress fiber assembly and reduces podosome prevalence. These findings suggest DDR1 is associated with shifts in cytoskeletal states in response to extracellular matrix stiffness.

Supporting this model, previous studies have shown fibroblasts retain mechanical memory of prior substrate stiffness, which can influence subsequent cytoskeletal and transcriptional responses [6,29]. Our data further indicate DDR1 can contribute to the regulation of integrin-associated cytoskeletal organization. DDR1 depletion impaired focal adhesion maturation and was associated with altered actin architecture, particularly in podosome-positive cells. Previous studies have implicated DDR1 in regulating actomyosin contractility and collagen contraction [30,31,32]. One possible mechanism is DDR1 could contribute to the stabilization of collagen-mediated adhesion complexes, potentially supporting force transmission and stress fiber assembly. Notably, we did not observe consistent or reproducible changes in canonical downstream signaling pathways, including RhoA, Rac1, Cdc42, Src, or FAK, following DDR1 depletion (Figure S3). This suggests DDR1-mediated effects are not mediated through classical signaling cascades but instead involve localized or context-dependent regulation of cytoskeletal and adhesion structures.

Although the molecular interface between DDR1 and integrin signaling remains to be elucidated, prior studies suggest context-dependent crosstalk between these pathways [33,34], supporting a role for DDR1 in integrin-dependent cytoskeletal regulation. Importantly, integrins remain the primary mediators of cell–matrix adhesion and mechanotransduction. However, our data show DDR1 depletion does not significantly alter global integrin β1 activation. In our functional assays, Itgb1 knockdown markedly impaired cell spreading and collagen remodeling, confirming the essential role of integrin β1 in these processes. These findings suggest DDR1 does not function as a primary regulator of integrin activation but instead modulates integrin-associated cytoskeletal organization in coordination with integrin-dependent pathways. This is consistent with the concept that DDRs function primarily as signaling receptors rather than direct mediators of cell adhesion. Notably, our functional assays demonstrate DDR1 not only regulates cytoskeletal organization but is also associated with changes in the mechanical and structural properties of the extracellular matrix. DDR1 depletion resulted in reduced collagen fibril stiffness, increased collagen degradation, impaired collagen fibril organization, and attenuated gel contraction. These observations further support an association between DDR1 and matrix stiffening and contractile remodeling, whereas its loss shifts fibroblasts toward a mechanically softened, matrix-degradative state.

Previous studies demonstrated DDR1 expression is upregulated in the renal interstitium following injury and has been implicated in fibrosis in vivo, supporting a potential role for DDR1 in matrix remodeling under pathological conditions. This mechanobiological interplay may contribute to the progression of fibrosis, in which increasing matrix stiffness contributes to the maintenance of contractile fibroblast phenotypes. This mechanobiological interplay could contribute to the progression of fibrosis, particularly in UUO-associated renal fibrosis, where activated fibroblasts progressively acquire matrix-producing and contractile phenotypes that promote extracellular matrix accumulation and tissue stiffening. The single-cell transcriptomic analysis demonstrated that Ddr1 expression was enriched in activated fibroblast populations, suggesting that DDR1 may be associated with fibroblast state transitions during fibrotic progression. Together with previous reports showing that DDR1 deficiency attenuates UUO-induced renal fibrosis, these findings support a potential role for DDR1 in maintaining contractile remodeling programs in activated fibroblasts. Interestingly, TGF-β response module scores were elevated during early fibroblast activation but were reduced in UUO 7 myofibroblasts. This pattern suggests that TGF-β signaling may primarily drive early transitional activation, whereas the maintenance of fully differentiated myofibroblasts may depend on alternative mechanisms. In this context, mechanically regulated pathways, including DDR1-associated signaling, may contribute to sustaining contractile and matrix-remodeling phenotypes in fibrotic tissues.

Several limitations should be acknowledged. The present study establishes associations between DDR1 expression, cytoskeletal organization, and matrix remodeling but does not define the direct molecular mechanisms underlying these relationships. In addition, our functional analyses were primarily conducted in vitro. Previous studies have demonstrated that DDR1 deficiency attenuates renal fibrosis in vivo, and DDR1 expression is upregulated in the renal interstitium following injury, such as in the UUO model. Although these studies primarily focused on infiltrating cells, the increased interstitial expression suggests that DDR1 may also be induced in mesenchymal compartments during fibrosis [20,35]; however, whether DDR1-dependent cytoskeletal remodeling directly contributes to fibrotic progression in vivo remains to be determined. Furthermore, fibroblast heterogeneity and additional mechanical properties of the extracellular matrix may further influence DDR1-associated responses and warrant future investigation.

In summary, we propose a model in which DDR1 modulates the balance between contractile and matrix-degradative remodeling states in renal fibroblasts. Rather than acting as a direct mechanosensor, DDR1 appears to influence integrin-associated cytoskeletal organization in response to biochemical and mechanical cues. In response to matrix stiffening, DDR1 is associated with stress fiber assembly, focal adhesion maturation, and contractile remodeling, whereas reduced DDR1 expression is associated with a shift toward a podosome-rich, matrix-degradative state.

## 4. Materials and Methods

### 4.1. Single-Cell RNA-Seq Analysis

Single-cell RNA sequencing data from murine kidneys subjected to sham operation, unilateral ureteral obstruction (UUO day 2 and UUO day 7), and reversed UUO (rUUO) were obtained from the Gene Expression Omnibus (GEO; GSE140023). Raw 10× Genomics count matrices were processed in R (v4.5.2) using Seurat (v5.4.0). Individual samples were read using Read10X, converted into Seurat objects, and merged into a single dataset. Cells expressing fewer than 200 genes, more than 7000 genes, or with mitochondrial transcript content exceeding 50% were excluded. Data were normalized using Seurat’s default LogNormalize method, and 3000 highly variable genes were selected for downstream analysis. Data were scaled, and principal component analysis (PCA) was performed. The first 30 principal components were used for graph-based clustering at a resolution of 0.6, and UMAP embedding was generated for visualization. Cell types were annotated using canonical marker gene sets and module scoring, followed by cluster-level assignment based on the predominant module-score identity. Fibroblasts were subsetted and reanalyzed using the same workflow, with 2000 highly variable genes, the first 20 principal components, and a clustering resolution of 0.6. Fibroblast transcriptional states were classified into Quiescent, Intermediate, and Myofibroblast states using curated quiescent, extracellular matrix, and contractile gene programs. Quiescent and contractile module scores were used to assign fibroblasts into three states, with cells not meeting the thresholds for either Quiescent or Myofibroblast classification assigned as Intermediate. Within each fibroblast state, differences in Ddr1 raw UMI counts between sham and injury/recovery conditions were assessed using pairwise Wilcoxon rank-sum tests with Benjamini–Hochberg correction. Raw UMI counts were used for quantitative visualization of Ddr1 expression. All analysis scripts used in this study are publicly available at: https://github.com/Po-YuChen/UUO-scRNA (accessed on 29 April 2026).

### 4.2. Cells and Reagents

Normal rat kidney fibroblasts (NRK-49F, RRID: CVCL_2144) were cultured at 37 °C in a humidified incubator with 5% CO_2_ and maintained in high-glucose Dulbecco’s Modified Eagle Medium (DMEM; Gibco, 12800-017, Thermo Fisher Scientific, Waltham, MA, USA) supplemented with 10% fetal bovine serum (FBS; Gibco, A5256701, Thermo Fisher Scientific, Waltham, MA, USA). For experimental treatments, cells were serum-starved in DMEM containing 1% FBS for 16 h prior to stimulation. Cells were treated with recombinant transforming growth factor-β1 (TGF-β1; PeproTech, 100-21C, PeproTech, Thermo Fisher Scientific, Waltham, MA, USA) at a final concentration of 10 ng/mL for 24 h unless otherwise indicated.

### 4.3. Stable Knockdown Clone Establishment

Stable DDR1 knockdown cell lines were generated using lentiviral short hairpin RNA (shRNA) constructs obtained from the RNA Technology Platform and Gene Manipulation Core Facility. The following constructs were used: scramble control (ASN0000000004), shDdr1 #1 (TRCN0000023371), and shDdr1 #2 (TRCN0000274558). Lentiviral particles were produced by co-transfecting HEK293FT cells with shRNA plasmids together with the packaging plasmids pΔ8.91 and pMDG using Lipofectamine 3000 (Invitrogen, L3000015, Invitrogen, Thermo Fisher Scientific, Waltham, MA, USA). After 24 h, the culture medium was replaced with DMEM containing 1% bovine serum albumin (BSA), and viral supernatants were collected 24 h later. Supernatants were clarified by centrifugation at 2000× *g* for 3 min and supplemented with polybrene (8 µg/mL) prior to infection. NRK-49F cells were infected with viral supernatants for 16 h. Following infection, cells were selected with puromycin (6 µg/mL) for at least 1 month and maintained in puromycin-containing medium thereby establishing stable knockdown cell lines for subsequent experiments. Knockdown efficiency was confirmed by Western blot analysis.

### 4.4. Western Blotting

Cells were lysed on ice in non-denaturing lysis buffer (20 mM Tris-HCl, 150 mM NaCl, 0.1% Triton X-100, 2 mM EDTA, 1 mM Na_3_VO_4_, 1 mM PMSF, and protease inhibitor cocktail). Lysates were clarified by centrifugation at 12,000× *g* for 10 min at 4 °C, and protein concentrations were determined using the DC™ Protein Assay (Bio-Rad, 5000116, Bio-Rad, Hercules, CA, USA). Equal amounts of protein (20 µg) were separated by SDS–PAGE using 7.5% or 10% gels and transferred to PVDF membranes by wet transfer. Membranes were blocked with 5% non-fat milk in TBST for 1 h at room temperature and incubated with primary antibodies overnight at 4 °C, followed by HRP-conjugated secondary antibodies for 1 h at room temperature. Signals were detected using enhanced chemiluminescence (ECL). β-actin was used as a loading control.

### 4.5. Immunofluorescence Staining

Cells were fixed with 4% paraformaldehyde for 10 min and permeabilized with 0.5% Triton X-100 for 5 min. Cells were then blocked with SuperBlock™ T20 (Thermo Fisher Scientific, Thermo Fisher Scientific, Waltham, MA, USA) for 1 h at room temperature without dilution. Cells were incubated with primary antibodies at a dilution of 1:1000–1:2000 overnight at 4 °C, followed by fluorophore-conjugated secondary antibodies for 1 h at room temperature. Nuclei were counterstained with DAPI or Hoechst 33258 (Sigma-Aldrich, St. Louis, MO, USA). Samples were mounted using an aqueous antifade mounting medium containing DAPI (Abcam, ab104139, Abcam, Cambridge, UK). Alternatively, samples were mounted using glycerol gelatin (Sigma-Aldrich, GG1) diluted 1:1 with PBS. Images were acquired using a Nikon ECLIPSE FN1 fluorescence microscope (Nikon Instruments Inc., Melville, NY, USA) or an Olympus FV3000 confocal microscope (Olympus, Tokyo, Japan).

### 4.6. Atomic Force Microscopy (AFM) Measurement of Collagen Fibril Stiffness

Collagen fibril stiffness was measured in fast force volume (FFV) mode using a sharp-tip cantilever (PFQNM-LC-A-CAL, Bruker; spring constant 0.1 N/m) mounted on a co-axial system combining an atomic force microscope (BioResolve, Bruker, Bruker, Berlin, Germany) and a laser scanning confocal microscope (FV-3000, Olympus, Tokyo, Japan). The cantilever was calibrated according to the manufacturer’s instructions prior to measurement. NRK-49F cells were cultured on FITC-labeled collagen gels for 5 days prior to measurement. Measurements were performed on living cells in culture medium over a scan area of 20 μm × 20 μm under controlled loading conditions. Force–distance curves were acquired across the scanned area, and local stiffness (Young’s modulus, kPa) was calculated by fitting the approach curves using the Sneddon contact model, assuming a conical tip geometry. The lateral resolution of the force map was approximately 100 nm. Stiffness values were extracted using AtomicJ software (version 2.3.1) and subjected to statistical analysis.

### 4.7. Collagen Aggregation Assay

FITC-conjugated type I collagen gels (final concentration 1 mg/mL) were prepared by neutralizing acid-solubilized collagen (3.33 mg/mL) with DMEM, NaHCO_3_, HEPES, CaCl_2_, and NaOH, followed by adjustment with culture medium to the desired volume. Cells were seeded into collagen gels and cultured for 24 h prior to TGF-β1 treatment. After 48 h of stimulation, cells were fixed with 4% paraformaldehyde and stained with TRITC-conjugated phalloidin to visualize F-actin. Fluorescence images were acquired using identical imaging settings across all conditions. Collagen aggregation was quantified by measuring FITC fluorescence intensity in regions outside the cell body using ImageJ. Cell boundaries were defined based on F-actin staining, and fluorescence intensity was normalized by subtracting background intensity measured from cell-free control gels.

### 4.8. Gel Contraction Assay

Cells were embedded in type I collagen gels (1 mg/mL, prepared as described above) and cultured for 24 h prior to TGF-β1 treatment. After 48 h of stimulation, gels were gently detached from the well edges to initiate floating gel contraction. Images were acquired at 0, 1, 2, 4, 8, and 24 h after gel release under identical imaging conditions. Gel area was quantified using ImageJ and expressed as a percentage of the initial gel area at the time of release. The initial gel area corresponded to the well area prior to contraction.

### 4.9. Protein Precipitation from Conditioned Medium

Conditioned medium was collected from cells cultured on collagen gels in DMEM containing 1% fetal bovine serum for 48 h. Supernatants were centrifuged at 2500× *g* for 5 min at 4 °C to remove cell debris. Proteins were precipitated by incubation with three volumes of pre-chilled acetone at −20 °C for 1 h. Precipitated proteins were pelleted by centrifugation at 13,000× *g* for 10 min, resuspended in 1× SDS sample buffer, and denatured by heating at 95 °C for 10 min prior to Western blot analysis. Equal loading was verified by Ponceau S staining of the membranes.

### 4.10. Polyacrylamide Gel Preparation

Glass coverslips (20 mm) were sequentially treated with 1 N NaOH for 10 min, silane (3-aminopropyltrimethoxysilane) for 10 min, and 0.5% glutaraldehyde to enable gel attachment. Polyacrylamide gels of defined stiffness (0.2, 2, and 20 kPa) were prepared by varying the ratios of acrylamide and bis-acrylamide, followed by polymerization using ammonium persulfate and tetramethylethylenediamine. Gels were activated with 2% EDC and 0.06% NHS in MES buffer to enable covalent coupling of type I collagen (50 µg/mL). Following collagen coating, gels were extensively washed with MES buffer and PBS, rinsed with culture medium, and equilibrated prior to cell seeding. Gel stiffness was validated by atomic force microscopy.

### 4.11. Podosome and Stress Fiber Quantification

Podosome structures were identified based on colocalization of cortactin and F-actin signals. Continuous cortactin/F-actin colocalized regions exceeding 10 µm^2^ were defined as podosome clusters. Cells containing at least one such cluster were classified as podosome-positive. Stress fiber-positive cells were defined as cells exhibiting aligned F-actin bundles spanning the cell body and occupying more than 50% of the cell area. Fluorescence images were acquired under identical imaging settings, and image analysis was performed using ImageJ. Thresholds were applied uniformly across all conditions. Quantification was conducted in a blinded manner from at least three independent experiments, with a minimum of 70 cells analyzed per condition.

## 5. Conclusions

In summary, this study identifies DDR1 as a regulator of cytoskeletal organization and matrix remodeling in renal fibroblasts. DDR1 expression is associated with a contractile cytoskeletal state and inversely correlates with podosome formation, highlighting its role in balancing contractile and matrix-degradative programs. Mechanistically, DDR1 promotes focal adhesion maturation and matrix stiffening while suppressing collagen degradation, thereby reinforcing a profibrotic microenvironment. These findings provide new insight into the mechanobiological regulation of fibroblast function and suggest DDR1 as a potential target for modulating fibrotic progression.

## Data Availability

The single-cell RNA sequencing dataset analyzed in this study is publicly available in the Gene Expression Omnibus (GEO) under accession number GSE140023. Processed datasets and analysis scripts are publicly available at: https://github.com/Po-YuChen/UUO-scRNA (accessed on 14 May 2026).

## Supplementary Materials

The following supporting information can be downloaded at: https://www.mdpi.com/article/10.3390/ijms27125419/s1.

## Abbreviations

The following abbreviations are used in this manuscript:

Acta2	Actin Alpha 2
AFM	Atomic Force Microscopy
COL1A1	Collagen Type I Alpha 1 Chain
Dcn	Decorin
DDR1	Discoidin Domain Receptor 1
DDR2	Discoidin Domain Receptor 2
ECM	Extracellular Matrix
FFV	Fast Force Volume
GEO	Gene Expression Omnibus
HEK293FT	Human Embryonic Kidney 293FT cells
Itgb1	Integrin Beta 1
Lum	Lumican
MMP2	Matrix Metalloproteinase 2
MMP9	Matrix Metalloproteinase 9
NRK-49F	Normal Rat Kidney Fibroblasts
PCA	Principal Component Analysis
Pdgfra	Platelet-Derived Growth Factor Receptor Alpha
Postn	Periostin
RRID	Research Resource Identifier
rUUO	Reversed Unilateral Ureteral Obstruction
scRNA-seq	Single-cell RNA sequencing
Tagln	Transgelin
TGF-β1	Transforming Growth Factor Beta 1
UMAP	Uniform Manifold Approximation and Projection
UMI	Unique Molecular Identifier
UUO	Unilateral Ureteral Obstruction
α-SMA	Alpha Smooth Muscle Actin
