# Supplementary material for: DDR1 Modulates Cytoskeletal Remodeling and Podosome Formation in Renal Fibroblasts
Source: Int J Mol Sci. 2026 Jun 16;27(12):5419. doi: 10.3390/ijms27125419 (PMC13300298; doi:10.3390/ijms27125419)
Supplement: Supplementary file 1 [file ijms-27-05419-s001.zip › ijms-4331139-supplementary.pdf]

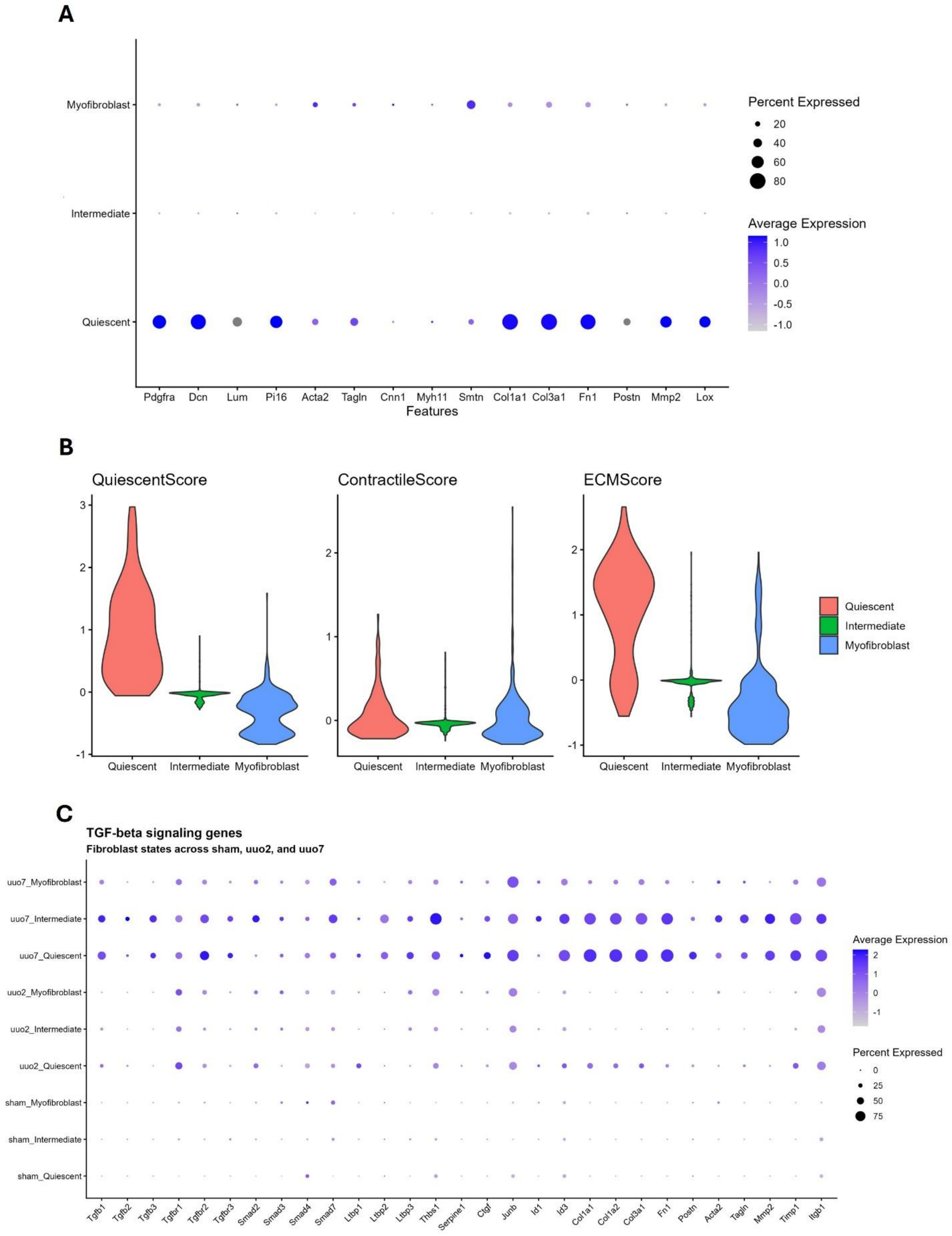
A
Myofibroblast
Intermediate
Quiescent
Pdgfra
Dcn
Lum
Pi16
Acta2
Tagln
Cnn1
Myh11
Smtn
Col1a1
Col3a1
Fn1
Postn
Mmp2
Lox
Features
Percent Expressed
20
40
60
80
Average Expression
1.0
0.5
0.0
-0.5
-1.0
B
QuiescentScore
ContractileScore
ECMScore
Quiescent
Intermediate
Myofibroblast
C
TGF-beta signaling genes
Fibroblast states across sham, uuo2, and uuo7
uuo7_Myofibroblast
uuo7_Intermediate
uuo7_Quiescent
uuo2_Myofibroblast
uuo2_Intermediate
uuo2_Quiescent
sham_Myofibroblast
sham_Intermediate
sham_Quiescent
Tgfb1
Tgfb2
Tgfb3
Tgfbr1
Tgfbr2
Tgfbr3
Smad2
Smad3
Smad4
Smad7
Ltbp1
Ltbp2
Ltbp3
Thbs1
Serpine1
Ctgf
Junb
Id1
Id3
Col1a1
Col1a2
Col3a1
Fn1
Postn
Acta2
Tagln
Mmp2
Timp1
Itgb1
Average Expression
2
1
0
-1
Percent Expressed
0
25
50
75

D

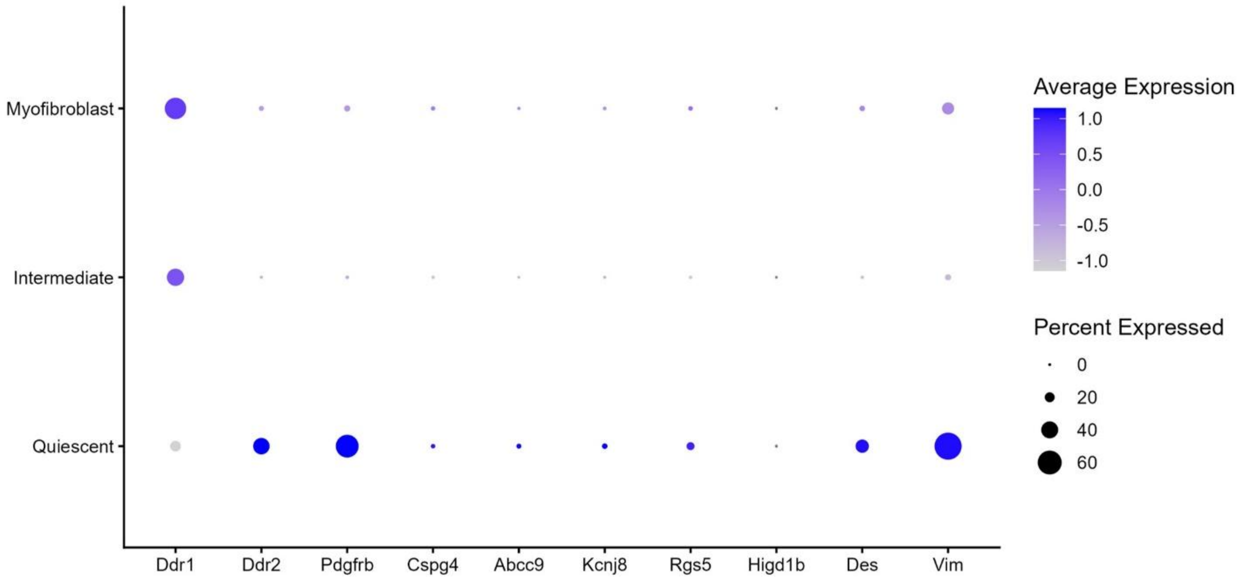

Figure S1. Validation of fibroblast state classification by marker gene expression and module scores. (A) Dot plot showing the expression of representative quiescent-, contractile-, and ECM-associated genes across the three fibroblast states (Quiescent, Intermediate, and Myofibroblast). Dot size indicates the percentage of cells expressing the gene within each state, and color intensity represents scaled average expression. (B) Violin plots of module scores used for fibroblast state classification. QuiescentScore, ContractileScore, and ECMScore were calculated based on predefined gene signatures and are shown across the three fibroblast states. The distribution of module scores supports the segregation of fibroblasts into Quiescent, Intermediate, and Myofibroblast states. (C) Dot plot of TGF-β signaling–related genes across fibroblast states and experimental conditions (sham, UUO 2, and UUO 7). Dot size represents the percentage of cells expressing each gene, and color intensity indicates average expression levels. Increased expression of TGF-β signaling genes is observed in intermediate and myofibroblast populations during UUO, with distinct state- and condition-dependent patterns. (D) Dot plot showing expression of Ddr1, Ddr2, pericyte/mural cell-associated markers (Pdgfrb, Cspg4, Abcc9, Kcnj8, Rgs5, Higd1b), and mesenchymal cytoskeletal markers (Des, Vim) across the three fibroblast states. Pericyte-associated markers were not broadly enriched in the intermediate or myofibroblast states, supporting that the current three-state fibroblast classification is not dominated by pericyte-like cells.

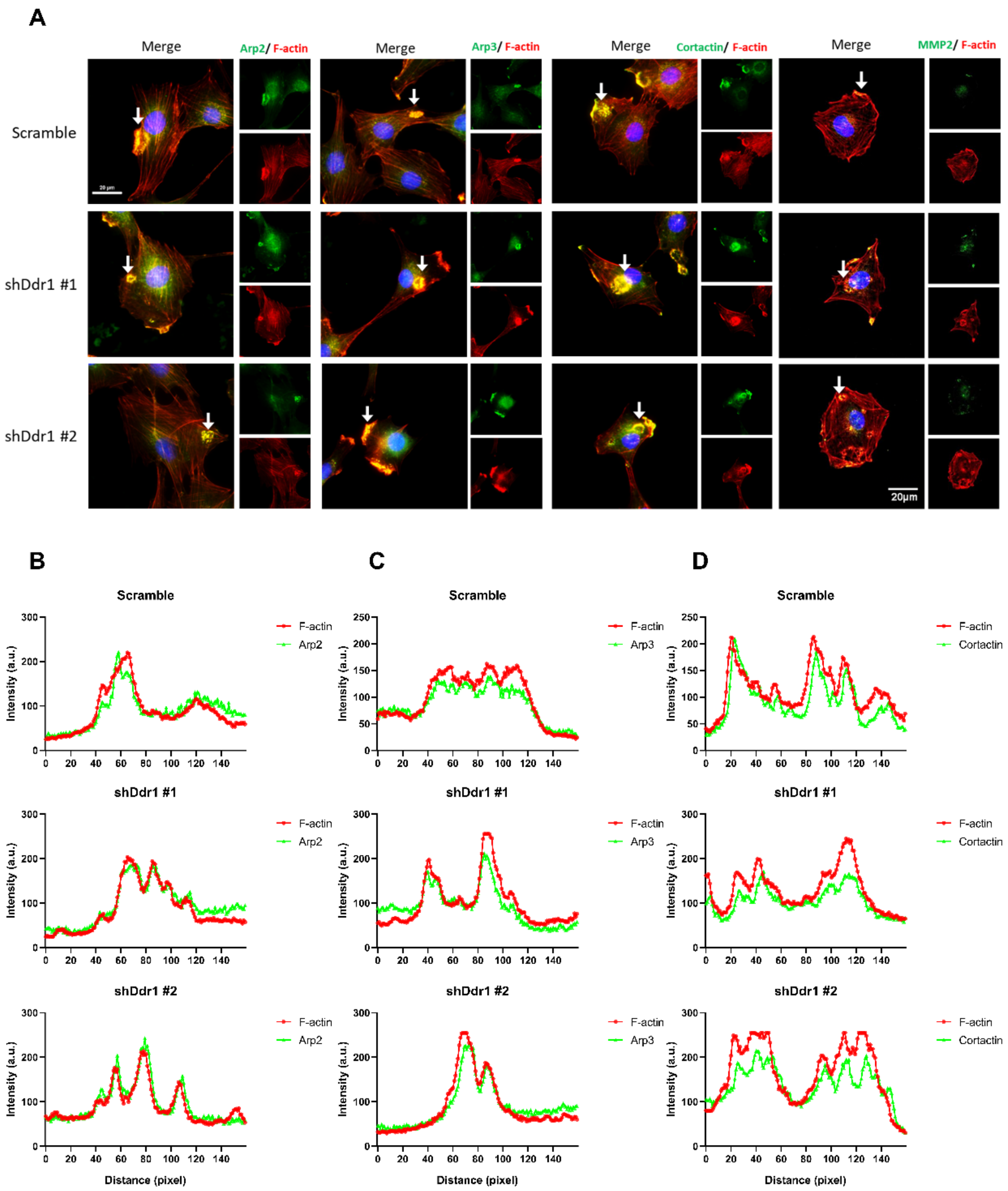

Figure S2. Quantification of actin aggregates as podosomes.

(A) Immunofluorescence staining of podosome markers (Arp2, Arp3, cortactin, and MMP2) in cells cultured for 24 h. White arrows indicate actin aggregates. (B–D) Quantitative colocalization analysis of podosome markers with F-actin.

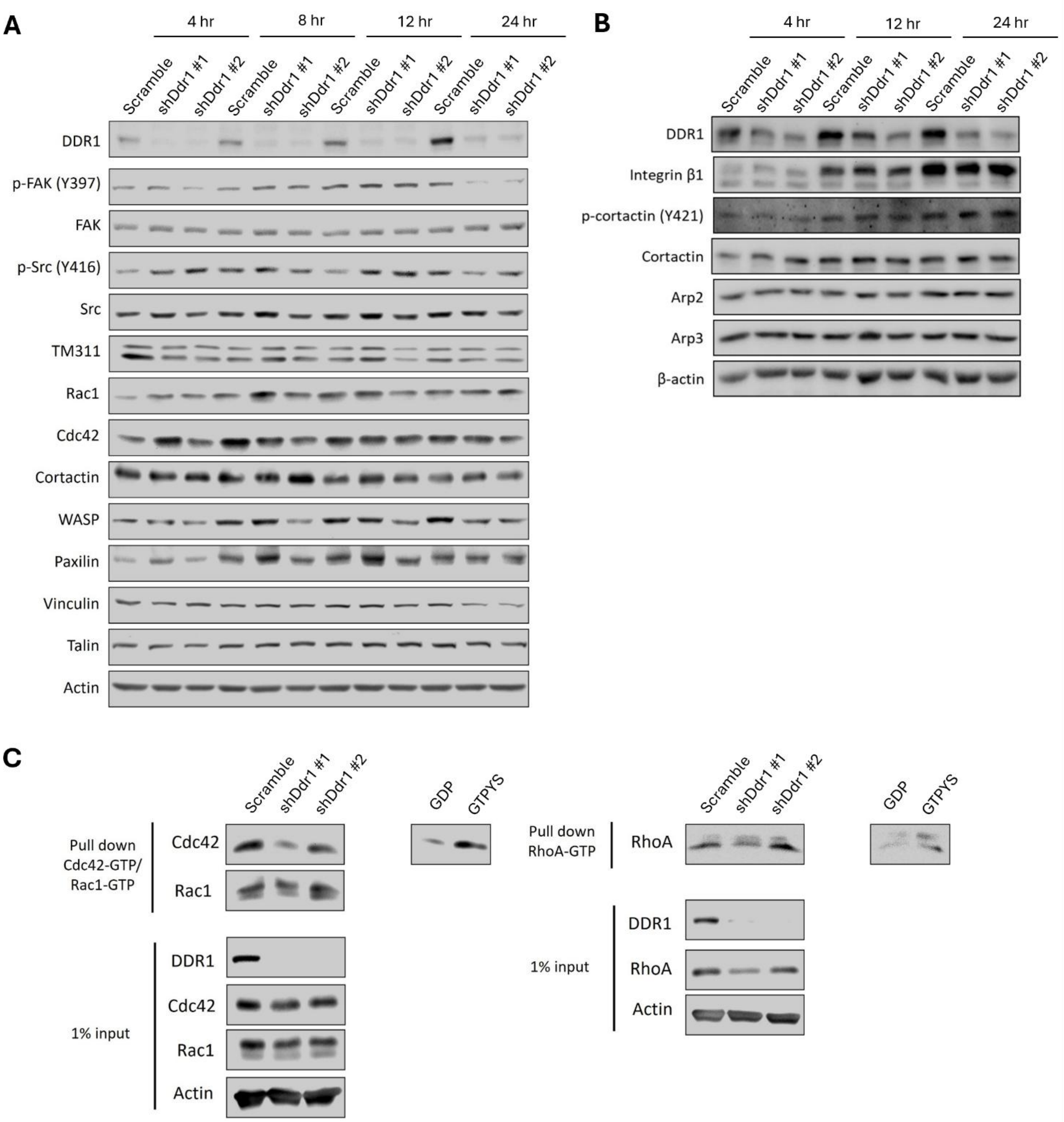

Figure S3. Analysis of signaling molecules, focal adhesion proteins, and small GTPase activity in DDR1-knockdown renal fibroblasts.

(A) Western blot analysis of focal adhesion- and cytoskeleton-associated proteins in Scramble, shDdr1 #1, and shDdr1 #2 NRK-49F cells seeded on tissue culture dishes for 4, 8, 12, and 24 h. Protein levels of DDR1, phospho-FAK (Y397), total FAK, phospho-Src (Y416), total Src, Tropomyosin (TM311), Rac1, Cdc42, cortactin, WASP, paxillin, vinculin, talin, and actin were examined. (B) Western blot analysis of integrin β1 and podosome-associated proteins in Scramble, shDdr1#1, and shDdr1#2 cells seeded on tissue culture dishes for 4, 12, and 24 h. Protein levels of DDR1, integrin β1, phospho-cortactin (Y421), total cortactin, Arp2, Arp3, and β-actin were analyzed. (C) Rac1/Cdc42 and RhoA activation assays were performed using commercially available pull-down assay kits (Cell Biolabs, STA-405). Active (GTP-bound) Rac1, Cdc42, and RhoA were analyzed in Scramble, shDdr1#1, and shDdr1#2 cells. GDP- and GTPγS-treated lysates were used

as negative and positive controls, respectively. Corresponding input lysates are shown below each assay.

	Clone ID	Target sequence
**Scramble (control)**	ASN0000000004	CCTAAGGTTAAGTCGCCCTCG
**shDdr1 #1**	TRCN0000023371	GCTGCTACTCTTGGTGACAAT
**shDdr1 #2**	TRCN0000274558	GATTCCACTTACGATGGATAT

Table S1. shRNA constructs used in this study

Antibody	Supplier	Application
DDR1 (5583S)	Cell Signaling Technology	WB
DDR2 (A01698-1)	Boster	WB
CD29 (610468)	BD Biosciences	WB
Active CD29 (553715)	BD Biosciences	WB
α-SMA (A5288)	Sigma	WB
β-actin (MAB1501)	Millipore	WB
Cortactin (H222)	Cell Signaling Technology	IF
Paxillin (610052)	BD Biosciences	IF
Arp2 (sc15389)	Santa Cruz Biotechnology	IF
Arp3 (sc48344)	Santa Cruz Biotechnology	IF
MMP2 (sc-13594)	Santa Cruz Biotechnology	IF
Collagen1a1 (PA21402)	Boster	WB
**Chemical**		
TGF-β1 (100-21C)	PEPROTECH	Cell treatment
Phalloidin (R415)	Invitrogen	IF

Table S2. Antibodies and reagents

Equipment/model	Brand	Application
SpectraMax® ABS Plus	Molecular Devices	Protein assay, Nucleotide quantification
iBright™ CL1500 Imaging System	Invitrogen	WB image
Microscope/ ECLIPSE FN1	Nikon	IF image
Microscope/ FLUOVIEW FV3000	Olympus	IF image

Table S3. Equipment used in this study
